# A Polydnavirus *Protein Tyrosine Phosphatase* Negatively Regulates the Host Phenoloxidase Pathway

**DOI:** 10.3390/v15010056

**Published:** 2022-12-24

**Authors:** Hong-Shuai Gao, Rong-Min Hu, Ze-Hua Wang, Xi-Qian Ye, Xiao-Tong Wu, Jian-Hua Huang, Zhi-Zhi Wang, Xue-Xin Chen

**Affiliations:** 1 Institute of Insect Sciences, College of Agriculture and Biotechnology, Zhejiang University, Hangzhou 310058, China; 2 Guangdong Laboratory for Lingnan Modern Agriculture, Guangzhou 510642, China; 3 Ministry of Agriculture Key Laboratory of Molecular Biology of Crop Pathogens and Insects, Zhejiang University, Hangzhou 310058, China; 4 Key Laboratory of Biology of Crop Pathogens and Insects of Zhejiang Province, Zhejiang University, Hangzhou 310058, China; 5 The Rural Development Academy, Zhejiang University, Hangzhou 310058, China

**Keywords:** protein tyrosine phosphatase, polydnavirus, phenoloxidase

## Abstract

Polydnavirus (PDV) is a parasitic factor of endoparasitic wasps and contributes greatly to overcoming the immune response of parasitized hosts. Protein tyrosine phosphatases (PTPs) regulate a wide variety of biological processes at the post-transcriptional level in mammals, but knowledge of PDV PTP action during a parasitoid–host interaction is limited. In this study, we characterized a PTP gene, *CvBV_12-6*, derived from *Cotesia vestalis* bracovirus (CvBV), and explored its possible regulatory role in the immune response of the host *Plutella xylostella*. Our results from qPCR show that *CvBV_12-6* was highly expressed in hemocytes at an early stage of parasitization. To explore *CvBV_12-6* function, we specifically expressed *CvBV_12-6* in *Drosophila melanogaster* hemocytes. The results show that Hml-Gal4 > *CvBV_12-6* suppressed the phenoloxidase activity of hemolymph in *D. melanogaster*, but exerted no effect on the total count or the viability of the hemocytes. In addition, the Hml-Gal4 > *CvBV_12-6* flies exhibited decreased antibacterial abilities against *Staphylococcus aureus.* Similarly, we found that *CvBV_12-6* significantly suppressed the melanization of the host *P. xylostella* 24 h post parasitization and reduced the viability, but not the number, of hemocytes. In conclusion, *CvBV_12-6* negatively regulated both cellular and humoral immunity in *P. xylostella*, and the related molecular mechanism may be universal to insects.

## 1. Introduction

Polydnaviruses (PDVs) are large double-stranded circular DNA (dsDNA) viruses that can be classified into bracoviruses (BVs) and ichnoviruses (IVs) according to their mutualistic relationship with braconid and ichneumonid wasps [1,2]. PDVs persist in a given wasp genome as proviruses with two distinct parts: structural core genes that are critical for the formation of virion particles, and proviral segments that serve as templates for the production of the virulence genes in its secondary host, primarily an insect in the Lepidoptera order [3]. These virulence genes alter host physiology, growth, and development, facilitating the survival of wasp offspring [1,4,5,6,7]. They are classified into gene families and contribute greatly to host immunosuppression and growth regulation [6,8,9]. Several BV virulence genes have been demonstrated to disrupt multiple elements of a host’s immune system. For example, two genes in *Microplitis demolitor* BV (MdBV), Egf 1.0 and Egf 1.5, which carry an epidermal growth-factor-like motif, have been demonstrated to inhibit melanization by targeting phenoloxidase-activating proteinase (PAP) [10,11]. Two members of the BV ankyrin gene family function as mimics of IκB proteins, and target the host nuclear factor-κB pathway to suppress the expression of antimicrobial peptides [12,13]. Recently, *Microplitis bicoloratus* bracovirus was found to induce apoptosis in host hemocytes by activating caspase-3 and suppressing the immune response by reducing the phosphorylation level of components of the pI3K/AKT signaling pathway [14,15]. Notably, the function of many other PDV genes is unknown.

In vertebrates, protein tyrosine phosphatases (PTPs), which catalyze the removal of the phosphate group from a tyrosine residue in a target protein, are indispensable and specific modulators of cellular signaling, thus regulating many processes, such as cell convergence and extension [16], cell differentiation [17,18,19,20], the signal transduction pathways in immunity [21,22,23], and metabolism-related pathways [24]. The structurally conserved PTP domain is key to membership in the PTP family, and three PTP groups have been categorized to date: (i) classical PTPs, (ii) PTPs with dual-specificity, and (iii) low-molecular-weight PTPs [25]. The PTP gene family has been found in the most extensively sequenced BV genome and constitutes the largest PDV gene family, except for the recently sequenced CinsBV of the *Chelonus insularis* wasp that does not contain PTP genes [26,27,28,29,30,31,32,33,34]. The BV PTP gene family expanded via classical mechanisms, including segmental duplication, tandem duplication, and dispersed duplication [35]. Bracovirus PTPs belong to the classical PTP family, and are characterized by their highly comparable PTP domain to vertebrate PTPs, with some carrying other functional domains [36]. Despite their sequence similarity, in contrast to classical PTPs, it is unclear whether the bracovirus PTPs are functional because they are highly divergent and lack conserved domains for substrate specificity, as proven by the fact that some BV PTPs are functional tyrosine phosphatases, whereas others are not [37,38]. Several BV PTPs have been demonstrated to suppress host immunity. For example, PTP-H2 and PTP-H3 of MdBV showed tyrosine phosphatase activity and involvement in the inhibition of phagocytosis by S2 cells [38]; Richard et al. [39] confirmed that the phosphatase activity of PTP-H2 in MdBV is critical for the induction of cell apoptosis. Hemocytes expressing PTP1 derived from *Cotesia vestalis* bracovirus (CvBV) exhibit a significant reduction in both cell spreading and encapsulation [40]. In addition, recent research has shown that a PTP from *Toxoneuron nigriceps* BV participates in the blockade of ecdysteroidogenesis, possibly by disrupting the phosphorylation balance of key proteins in the MAPK and PI3K ecdysone pathways, altering host growth and development [41,42]. However, the role of most BV PTPs in host–parasitoid interactions has not been well elucidated.

*Cotesia vestalis* (Haliday) is a major natural enemy of *Plutella xylostella* (L.) (Lepidoptera: Plutellidae), a destructive pest of Brassica crops worldwide [43]. Our previous studies showed that the CvBV genome is composed of 30 DNA segments that encode 218 genes in 13 gene families, with CvBV-PTP constituting the largest gene family (with 33 PTP genes) [44]. In this study, we focused on characterizing the immunosuppressive role of CvBV-PTP genes. We found a PTP gene, designated *CvBV_12-6*, encoded by the 6th gene in the 12th segment of the CvBV genome. CvBV_12-6 was verified to show phosphatase activity. Ectopic expression of *CvBV_12-6* in the *D. melanogaster* model suppressed the phenoloxidase (PO) activity of hemolymph and increased the susceptibility to *Staphylococcus aureus*. Furthermore, we found that *CvBV_12-6* inhibited cellular immunity and humoral immunity in the *P. xylostella* host by reducing the hemocyte viability and suppressing the PO activity in the host hemolymph.

## 2. Materials and Methods

### 2.1. Insect Rearing

*Plutella xylostella* and its endoparasitoid *Cotesia vestalis* were reared as previously described [45]. They were maintained in a 14 h light/10 h dark photoperiod at 25 ± 1 °C with 65% relative humidity. Adult *P. xylostella* and *C. vestalis* were fed a 20% honey/water (V/V) solution. To ensure a high parasitization rate, middle third instar host individuals were exposed to a single female wasp within a test tube until oviposition was observed. W1118 wild-type and Bloomington Hml-GAL4 (BDSC_30140) *D. melanogaster* stock was used in this study, and the flies were reared on standard cornmeal/yeast/agar medium at 18 °C.

### 2.2. Sequence Analysis

According to the annotated CvBV genome, the sequences of *CvBV_12-6* were downloaded. The signal sequence was predicted by SignalP 6.0 (https://services.healthtech.dtu.dk/service.php?SignalP, accessed on 2 January 2021). Multiple sequence alignment was performed with DNAMAN (V10.0.2.128) software.

### 2.3. RNA Extraction and ORF Cloning

RNA was extracted from five *P. xylostella* larvae at 12 h, 24 h, 48 h, and 96 h post parasitization (pp), respectively. TRIzol reagent (Invitrogen, Carlsbad, CA, USA) was used to isolate total RNA according to the manufacturer’s instructions. RNA purity was determined with a NanoDrop^®^ spectrophotometer (Thermo Fisher, Waltham, MA, USA). A SuperScriptTM III Reverse Transcriptase kit (Vazyme, Nanjing, China) was used to synthesize a complementary DNA (cDNA) library. The primers used for PCR are shown in Table 1. PCR was performed under the following cycling conditions: 94 °C for 2 min, 35 cycles of 95 °C for 30 s, 60 °C (according to different primers inducing change) for 30 s, and 72 °C for 15 s, and then 72 °C for 10 min. Each PCR product was cleaned with a Wizard^®^ SV Gel and PCR Clean-Up System (Promega, Madison, WI, USA) kit, and then cloned into a pGEM-T Easy Vector. Recombinant vectors were transferred into the *E. coli* TG1 strain, and positive clones were sequenced by Sangon (Shanghai, China). The sequenced fragments were assembled by SeqMan software (DNASTAR, 7.1.0.44 version), and then aligned with the CvBV genome with BLASTP (http://www.ncbi.nlm.nih.gov/, accessed on 2 February 2021).

### 2.4. Expression Analyses by qPCR

To analyze gene expression levels in different developmental stages, five *P. xylostella* larvae were collected as RNA samples 6 h, 12 h, 24 h, 48 h, 72 h, 96 h, and 120 h after parasite exposure. qPCR was performed as previously described [46]. A ReverTra Ace qPCR RT kit (Toyobo, Osaka, Japan) was used to synthesize cDNAs. The primers used for qPCR are shown in Table 1. *β*-*Tubulin* (GenBank accession No. EU127912) from *P. xylostella* was used as the reference gene. qPCRs were performed on a CFX Connect real-time system (Bio–Rad, Hercules, CA, USA) using THUNDERBIRD qPCR Mix (Toyobo, Osaka, Japan). Each qPCR for a given gene and time point was performed using at least three biological replicates under the following cycling conditions: 95 °C for 60 s, 40 cycles of 95 °C for 15 s, and 60 °C for 30 s. The results were analyzed via the 2^-ΔΔCT^ method.

Similarly, to detect the tissue preference of *CvBV_12-6* in a host, hemocytes and tissues, i.e., central nervous system (CNS), fat body, midgut, cuticle, testis, silk gland, and malpighian tubule tissues, from different developmental stages of parasitized larvae (30 larvae per sample) were dissected in phosphate-buffered saline (PBS) and then subjected to qPCR. The qPCR program was the same as that described above. All assays were performed at least three times.

### 2.5. Recombinant Baculovirus Concentration

The pFASTBAC-HTb (Invitrogen, San Diego, CA, USA) vector for baculovirus expression in *P. xylostella* hemocytes was modified by inserting the open reading frame (ORF) sequence of *CvBV_12-6* with an HA tag using conventional molecular biology techniques. The GFP gene was used as the negative control, and the sequence was cloned from a pRSET-eGFP vector (Thermo, USA, V35320). The primers used are shown in Table 1. Recombinant nucleopolyhedroviruses (NPVs), NPV-*CvBV_12-6* or NPV-*GFP*, were produced with a Bac-to-Bac Baculovirus Expression System (Invitrogen, San Diego, CA, USA) according to the manufacturer’s instructions. The recombinant NPVs were concentrated by centrifugation as described by [47] and then resuspended in 100 μL of PBS buffer (pH 7.4). The titer of the generated high-titer virus stock was determined by viral plaque assay according to the Bac-to-Bac Baculovirus Expression System manufacturer instructions (Invitrogen, San Diego, CA, USA). An overexpression experiment was performed by injecting 1 × 10^5^ copies of NPV-*CvBV_12-6* or NPV-*GFP* into third instar *P. xylostella* larvae.

### 2.6. Tyrosine Phosphatase Assay and Western Blotting

For convenient protein collection, the recombinant baculovirus NPV-*CvBV_12-6* and NPV-*GFP* (5 × 10^7^ copies) were expressed in Sf9 cells (5 × 10^6^ cells), which were cultured at 27 °C in a cell incubator. All the cells were collected 2 days later and washed with PBS at least three times. The clean cells were lysed with a reagent (Promega, Madison, WI, USA), and the following procedure was performed according to the instructions of a tyrosine phosphatase assay kit (Promega, Madison, WI, USA). The activity of tyrosine phosphatase depends on the concentration of free phosphate, which can be reflected by the absorbance read at 630 nm.

The recombinant CvBV_12-6 protein from Sf9 cells was detected by western blot using an HA monoclonal antibody (Sangon, Shanghai, China), and an anti-Actin monoclonal antibody (Sangon, Shanghai, China) was used as the internal control. Samples were diluted in 5× protein sodium dodecyl sulfate–polyacrylamide gel electrophoresis loading buffer (Sangon, Shanghai, China) and then boiled for 10 min. The proteins in the samples separated in the denaturing polyacrylamide gel were transferred to a polyvinylidene difluoride membrane. After blocking and washing, the membranes were incubated with primary antibodies against HA or actin (1: 10,000) for 2 h at room temperature. The membranes were then incubated with horseradish peroxidase-conjugated goat anti-mouse IgG secondary antibody diluted 1:3000 in Tris-buffered saline with Tween-20. After washing five times, the membranes were incubated with enhanced chemiluminescence western blot substrate (Promega, Madison, WI, USA).

### 2.7. Transgenic Fly Construction

The GAL4/UAS (upstream activator sequence) binary expression system was used to study the function of *CvBV_12-6* in *D. melanogaster* [48]. The open reading frame (ORF) sequence of *CvBV_12-6* was cloned into a pUAST-attb vector [49]. A transgenic fly carrying the UAS-*CvBV_12-6* gene was obtained by phiC31 integrase-mediated insertion into the attP2 landing site locus on the third chromosome. We used Hml-GAL4 (BDSC_30140), an expression driver of lymph glands and circulating hemocytes, to drive *CvBV_12-6* expression in the *D. melanogaster* hemocytes [50]. Thirty adult Hml-GAL4 strain males and 200 adult UAS-*CvBV_12-6* strain virgins were selected, mated for 1 day, and transferred to a new food bottle every 2 h. Larvae from crosses of Hml-GAL4 and W1118 were used as controls.

### 2.8. RNA Interference

Double-stranded RNA (dsRNA) specific for *CvBV_12-6* and *GFP* was produced and purified using a T7 RiboMAXTM Express kit (Promega, Madison, WI, USA). The primers used for dsRNA synthesis are shown in Table 1. The synthesis products were confirmed by running dsRNA on an agarose gel; before parasitization, 1 µg of *CvBV_12-6* or *GFP* dsRNA was injected into third instar *P. xylostella* larvae using an Eppendorf Femto jet device (Eppendorf, Germany). The efficiency of the interference was determined individually by qPCR 12 h, 24 h, 36 h, and 48 h pp.

### 2.9. Hemocyte Count

The hemolymph of *P. xylostella* was collected 24 h after RNAi or injection of NPVs. A trypan blue stock solution was diluted to generate a 1× work solution, and 19.5 µL of the trypan blue work solution was mixed with 0.5 μL of hemolymph. This reagent was then added to a cell counting plate, and Countstar 1.0 software was used to determine the cell number and viability automatically using 6–15 μm as the initial detection parameter.

### 2.10. PO Activity Assays

PO activity was measured as previously described [51] with a minor modification to the procedure described by Goldsworthy et al. [52]. Approximately 5 μL of hemolymph from approximately 30 *P. xylostella* larvae 24 h post treatment was added to 95 μL of anticoagulant buffer (100 mmol/L glucose; 62 mmol/L NaCl; 30 mmol/L trisodium citrate; 26 mmol/L citric acid, and 10 mmol/L ethylenediaminetetraacetic acid, pH 4.6). To remove the hemocytes, the diluted hemolymph was centrifuged at 1000× *g* for 5 min. A Better Bradford AssayTM kit (Thermo, USA) was used to determine the protein concentration of each diluted cell-free hemolymph sample. Subsequently, 20 µL of heat-killed *Micrococcus luteus* (optical density (OD) = 0.5) was added to the diluted hemolymph and incubated for 20 min at room temperature. The mixture was then used to immediately measure the PO activity. PO activity was measured spectrophotometrically by recording the formation of dopachrome from L-dihydroxyphenylalanine (L-DOPA). Each assay was established in a 96-well plate with a total volume of 200 μL (with 140 μL of L-DOPA [3 g/L] and 60 μL of diluted cell-free hemolymph). The absorbance at 490 nm was measured once every 5 min for a total of 60 min. PO activity is expressed as a change in absorbance at 490 nm per mg protein per min. Each assay was performed with at least three biological replicates.

As for *D. melanogaster*, 30 adult Hml-GAL4 strain males and 200 adult UAS-*CvBV_12-6* or W1118 strain virgins were crossed and laid eggs in different bottles. The offspring in one bottle were used as a replicate. The hemolymph was collected from approximately 30 third instar offspring larvae and PO activity was measured as described above. The assay was replicated three times.

### 2.11. Survival Assay

A survival assay was performed as previously described [53]. *Staphylococcus aureus* is a gram-positive bacteria that is among the most frequent cause of morbidity and mortality due to infection worldwide [54]. The strength of the immune response of *D. melanogaster* larvae was measured to determine the risk of *S. aureus* infection. *S. aureus* was grown overnight at 37 °C with shaking at 250 r/min. Cultures were centrifuged at 1000× *g* for 1 min. The bacteria were resuspended in sterile PBS until an OD_600_ of 0.4 was obtained. Seven days post eclosion, a male fly was injected with 40 nL of bacterial resuspension using an Eppendorf Femto jet device (Eppendorf, Germany) with a microcontroller (Narishige, Japan). Experiments were performed in triplicate (n = 20 flies per treatment). Injections were performed under a Stemi 2000-C microscope (Zeiss, Germany). Flies were reared at 25 °C after injection. Flies that died within 6 h were considered dead and removed from the final count. Flies were transferred every day to provide new food sources, and fly death was recorded every 12 h for 3 days.

### 2.12. Statistical Analysis

All the data were calculated as the mean ± SD. The significant difference between samples was determined by one-way analysis of variance (ANOVA) with Tukey’s test, and the significance threshold was set to a *p* value < 0.05. Log rank tests were performed to determine whether the survival curves between different treatment groups were significantly different.

## 3. Results

### 3.1. Characterization of CvBV_12-6

**Figure 1:**
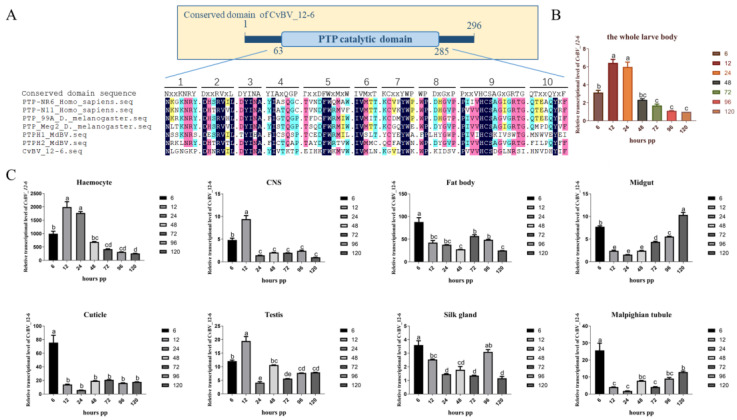
The conserved motifs and expression patterns of *CvBV_12-6*. (**A**) Image showing the conserved domain in the CvBV_12-6 protein and the amino acid sequence of each motif. X represents any amino acid. Conserved domain sequence: PTP NR6 and PTP N11 were from *Homo sapiens*; PTP_meg2 and PTP_99A were from *D. melanogaster*; PTP H1 and PTP H2 were from MdBV. (**B**) The transcriptional dynamics of *CvBV_12-6* in *P. xylostella* 6, 12, 24, 48, 72, 96, and 120 h post parasitization (pp). (**C**) The expression pattern of *CvBV_12-6* in different tissues of parasitized *P. xylostella.* Data are presented as the mean ± SD based on three independent experiments. Differences among samples were tested via Tukey’s test, and different letters indicate significant differences at a *p* value < 0.05.

The ORF of *CvBV_12-6* (GenBank accession No. QZB49121.1) was 891 base pairs (bp) and encoded 296 amino acids (aa), and the molecular weight of the protein was predicted to be 34.12 kDa. A PTP catalytic domain (Figure 1A) in CvBV_12-6 was identified, and it showed no transmembrane domain or signal peptide, indicating that it acted intracellularly. Multiple sequence alignment showed that CvBV_12-6 carries ten motifs, and most of these motifs differed from those in classical vertebrate PTPs, except for Motif 2 and Motif 3 (Figure 1A) [44]. Although four residues in Motif 9 (D9, N12, S14, and I15) differed from the conserved sequence, the conserved catalytic site with cysteine (C6) in Motif 9 suggests that this PTP was a functional phosphatase, as described in a previous paper [37].

### 3.2. Expression Analyses of CvBV_12-6 in P. xylostella

To determine the expression pattern of *CvBV_12-6*, we determined the expression levels of *CvBV_12-6* in parasitized *P. xylostella* larvae at different developmental stages and in different tissues via qPCR. Our analysis shows that the transcriptional level of *CvBV_12-6* peaked 12 h post parasitization (pp) and then dropped gradually 24 h pp (Figure 1B). The expression of *CvBV_12-6* in different tissues showed that *CvBV_12-6* was expressed in all the tested tissues, with the highest transcript abundance in hemocytes and the second highest in the fat body. The expression level in the hemocytes at 12 h pp was at least 40-fold higher than that in the fat body, buts its transcriptional level in the other six tissues was relatively low at each sampling time point (Figure 1C).

### 3.3. The CvBV_12-6 Protein Exhibited Tyrosine Phosphatase Activity

**Figure 2:**
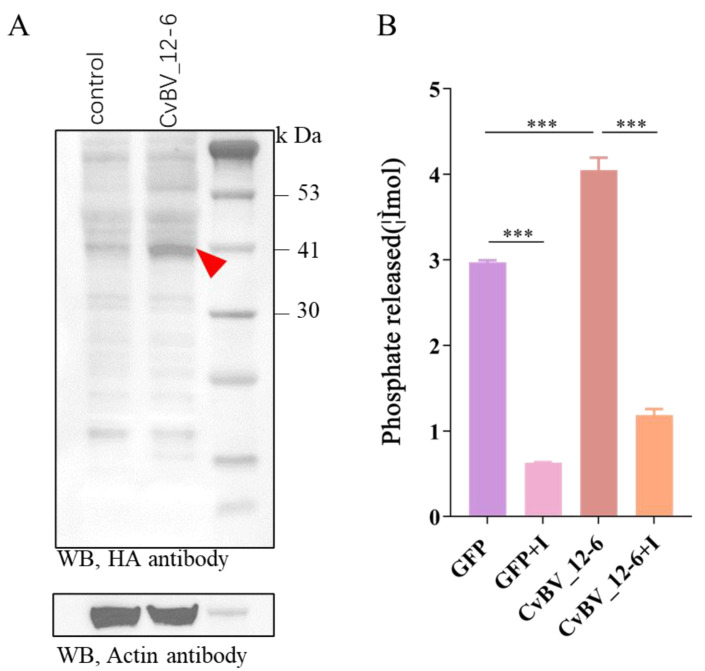
Eukaryotic expression of CvBV_12-6 and tyrosine phosphatase activity detection. (**A**) The expression of CvBV_12-6 in the Sf9 cell line was detected by western blotting using an HA tag monoclonal antibody, and actin was used as the internal control. (**B**) The tyrosine phosphatase activity of CvBV_12-6 in Sf9 cells was measured by the amount of phosphate released after the incubation of the total protein and synthesized phosphopeptides. The cell lysate was extracted 48 h post infection. The GFP was used as the negative control, and sodium vanadate was used as the protein tyrosine phosphatase inhibitor (I). Data are presented as the mean ± SD. Differences among samples were evaluated for significance via Tukey’s test (***, significant difference at a *p* value < 0.001).

To determine whether CvBV_12-6 was functional, we collected Sf9 cells infected with the recombinant NPV-*CvBV_12-6* for 2 days and then extracted the total proteins. Compared with the uninfected Sf9 cells, western blot results show a clear band with a corresponding protein size of 40 kDa, which was slightly larger than the predicted size of CvBV_12-6 (34.12 kDa) (Figure 2A). Compared with the NPV-*GFP* group, the cells that expressed *CvBV_12-6* released more free phosphate, indicating that *CvBV_12-6* showed tyrosine phosphatase activity (Figure 2B).

### 3.4. CvBV_12-6 Negatively Regulates the Phenoloxidase Pathway in D. melanogaster

**Figure 3:**
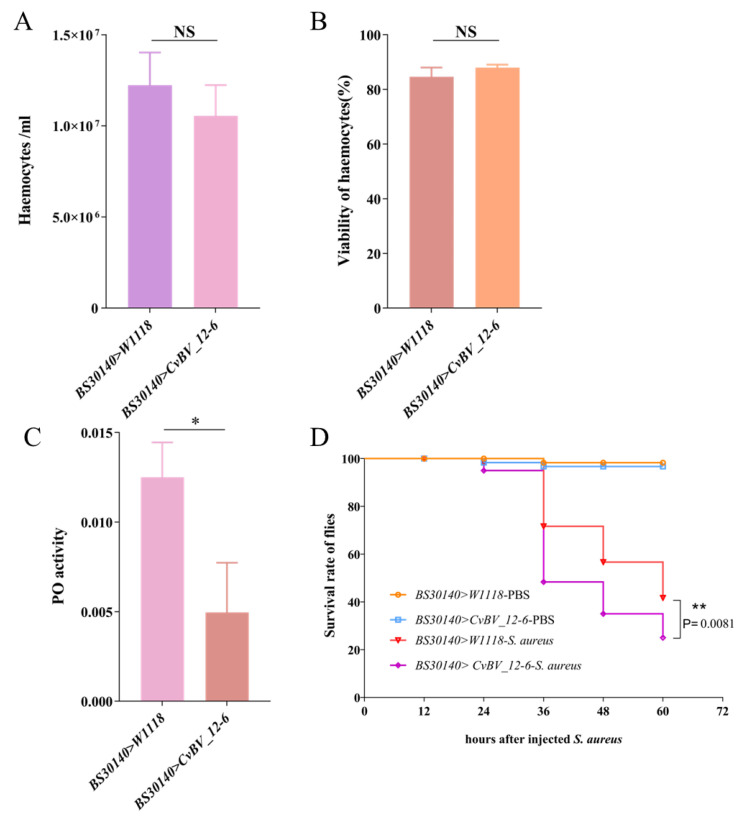
The immunosuppression of *CvBV_12-6* in *D. melanogaster*. (**A**,**B**) The effect of *CvBV_12-6* on the number (**A**) and the viability of hemocytes (**B**) in *D. melanogaster*. (**C**) The effect of *CvBV_12-6* on the PO activity in *D. melanogaster*. The hemolymph was collected from third instar *D. melanogaster* larvae, and the PO activity was determined as a change in absorbance at 490 nm per mg protein per min. (**D**) The antibacterial activity of male *D. melanogaster* to *S. aureus.* Thirty adult Hml-GAL4 strain males and 200 adult UAS-*CvBV_12-6* strain virgins were mated for 1 day and then transferred to a new food bottle every 2 h. Hml-GAL4 flies crossed with W1118 flies were the controls. Data are presented as the mean ± SD from three independent experiments. Each treatment group included 20 larvae (n = 15). Differences among samples were evaluated for significance via Tukey’s test (NS, no significance; *, significant difference at a *p* value < 0.05; **, significant difference at a *p* value < 0.01).

In contrast with those in Hml-Gal4 > W1118 flies, the total count and viability of hemocytes in third instar Hml-Gal4 > *CvBV_12-6 D. melanogaster* larvae were not affected (Figure 3A,B). We measured the PO activity of hemolymph in the *D. melanogaster* larvae. The PO activity of hemolymph in Hml-Gal4 > *CvBV_12-6* larvae was significantly lower compared with that in Hml-Gal4 > W1118 flies (Figure 3C), suggesting a suppressive role for *CvBV_12-6* during melanization. In addition, Hml-Gal4 > *CvBV_12-6* flies showed increased susceptibility against *S. aureus* infection (Figure 3D), suggesting a decrease in antibacterial ability due to the expression of *CvBV_12-6*.

### 3.5. CvBV_12-6 Decreases the Viability of Hemocytes in the P. xylostella Host

**Figure 4:**
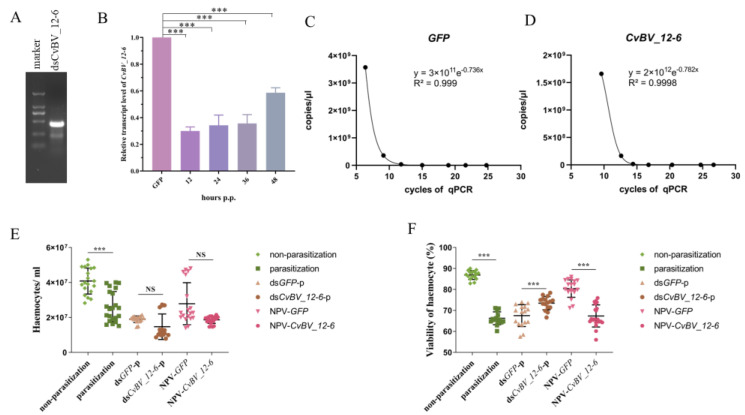
The effect of *CvBV_12-6* on hemocytes of the *P. xylostella* host. (**A**) The biosynthesis of dsRNA of *CvBV_12-6* was detected by agarose gel electrophoresis. (**B**) The RNA interference efficiency was measured by qPCR. (**C**,**D**) A standard curve was used to calculate the titer of the recombinant baculovirus as determined by qPCR. (**E**,**F**) The effect of *CvBV_12-6* on the hemocyte count and the hemocyte viability in *P. xylostella*. The hemolymph of *P. xylostella* was collected 24 h after parasitization, RNAi or injection of NPV. Then, 19.5 µL of trypan blue working solution was mixed with 0.5 μL hemolymph, and then added to a cell counting plate. Count Star 1.0 software was used to automatically analyze the cell number and viability. ds*CvBV_12-6*-p: ds*CvBV_12-6* was injected into third instar larvae, and parasitization was performed immediately; ds*GFP*-p was used as the negative control. NPV-*CvBV_12-6*: injection of NPV-*CvBV_12-6* into third instar larva; NPV-*GFP* was used as the negative control. Each treatment group included more than 30 larvae (n >15). Data are presented as the mean ± SD. Differences among samples were evaluated for significance via Tukey’s test (***, significant difference for a *p* value < 0.001).

Considering the results with flies, we explored the immunosuppressive role played by CvBV_12-6 in its natural host, *P. xylostella*. The expression level of CvB*V_12-6* was reduced by the injection of gene-specific dsRNA. Our results show that the relative expression level of *CvBV_12-6* was decreased by at least 40% 48 h after dsRNA injection (Figure 4A,B). Moreover, we overexpressed *CvBV_12-6* in *P. xylostella* by injecting recombinant NPV-*CvBV_12-6*. The injection dosage was calculated based on the standard curve (Figure 4C,D). Then, we injected 1 × 10^5^ copies of recombinant baculoviruses according to the protocol of a Bac-to-Bac system; however, this concentration was not sufficient for infecting insect cells.

Compared with those of normal *P. xylostella* larvae, the total count and the viability of hemocytes were significantly decreased in parasitized hosts (Figure 4E,F). In addition, *CvBV_12-6* played a negative role in the viability of hemocytes, but exerted no effect on the total hemocyte count. Compared to the control group, the knockdown of *CvBV_12-6* significantly increased the viability of *P. xylostella* hemocytes, while the overexpression of *CvBV_12-6* by recombinant baculovirus injection significantly decreased the viability compared with that of the control baculovirus injection (Figure 4F).

### 3.6. CvBV_12-6 Inhibits Melanization of P. xylostella

**Figure 5:**
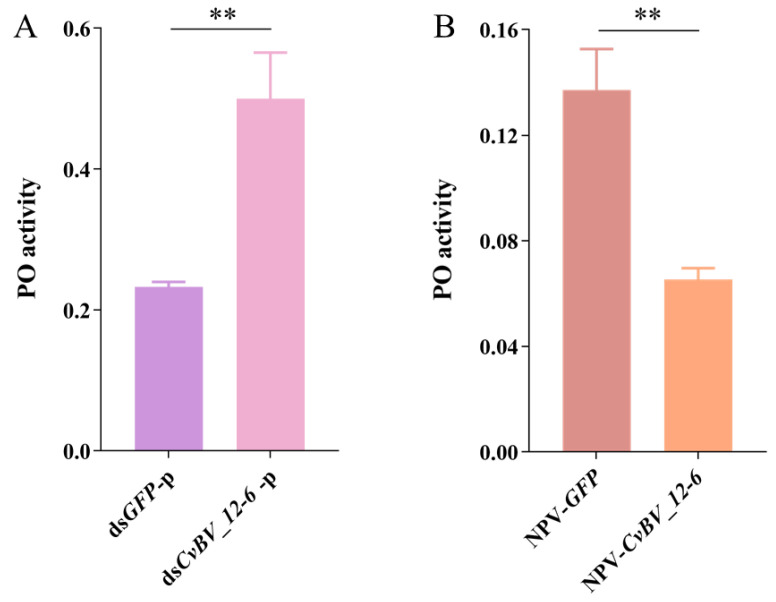
*CvBV_12-6* inhibited the melanization response of the *P. xylostella* host. (**A**) One microgram of ds*CvBV_12-6* or ds*GFP* was injected into third instar *P. xylostella* larvae, and parasitization was performed immediately. The hemolymph was collected, and the PO activity was measured 24 h pp. (**B**) NPV-*CvBV_12-6* or NPV-*GFP* was injected into third instar *P. xylostella* larvae, and the PO activity was measured 24 h post injection. Data are presented as the mean ± SD. Differences among samples were evaluated for significance via Tukey’s test (**, significant difference at a *p* value < 0.01).

In *D. melanogaster*, we found that *CvBV_12-6* effectively suppresses the PO pathway; therefore, we hypothesized that its function in *P. xylostella* is the same. Considering the higher expression level of *CvBV_12-6* 12 h and 24 h pp and the parasitization by *C. vestalis* significantly suppressing PO activity of the host hemolymph 24 pp in previous research [55], we measured the PO activity of *P. xylostella* hemolymph 24 pp. As shown in Figure 5A, the PO activity was significantly increased at 24 h pp in the host larvae treated with ds*CvBV_12-6*. Consistent with that in parasitized hosts, the PO activity in the host hemolymph was significantly suppressed 24 h after *CvBV_12-6* was initially overexpressed (Figure 5B).

## 4. Discussion

Polydnavirus-mediated disruption of cellular and humoral immunity renders parasitized lepidopteran larvae suitable for the development of wasp larvae as well as makes them susceptible to opportunistic infections [8]. In the *P. xylostella*–*C. vestalis* system, a series of PDV genes are delivered into the host genome to inhibit the host immune response and contribute greatly to successful parasitization [6,8,9]. In this study, we characterized a PTP gene, *CvBV_12-6*, encoded by CvBV, and explored its role in the immune response of the host.

Previous research had identified 10 conserved motifs in 113 vertebrate PTP domains, and these structural motifs were shown to work together to regulate the dephosphorylation process precisely [36]. The proposed role of each motif during dephosphorylation has been well characterized: Motifs 2 to 7 play key roles in stabilizing the secondary structure of the PTP domain; Motifs 1 and 8 are required for substrate recognition; Motifs 9 and 10 participate in substrate binding, catalysis, and optimal hydrolysis, respectively [36,37]. In our research, multiple sequence alignment showed that *CvBV_12-6* carries ten motifs, with most diverging from the motifs of classical vertebrate PTPs, except for Motif 2 and Motif 3 (Figure 1A). We speculate that this divergence probably evolved in the context of parasitism, which allowed parasites to recognize different substrates or similar substrates in different host species. In addition to this study, other BV PTPs, in which the motifs divergence from the classical motifs, have been demonstrated to exhibit tyrosine phosphatase activity; these PTPs include PTP-H2 and PTP-H3 from MdBV [38]. Notably, while four residues (D9, N12, S14, and I15) in Motif 9 of *CvBV_12-6* differ from those at these sites in classical PTPs, conservation of the cysteine (C6) site suggests that the PTP functions as a phosphatase, as described in previous papers [37,56,57]. This evidence suggests that whether bracovirus PTPs exhibit phosphatase activity does not entirely depend on the conservation of the motifs, but on the conservation of some important amino acid residues.

The expression of *CvBV_12-6* in different tissues reveals that *CvBV_12-6* was expressed in all the tested tissues with the highest transcript abundance in the hemocytes, which are critical for many immune responses in insects [58]. Hence, we infer that enriching *CvBV_12-6* in hemocytes may have modulated the host immune response. As an effective system to study proteins of unknown function, the *D. melanogaster* model has been employed in the study of the function of BV virulence genes [59], and we used this model in our study. As a nonmodal organism, no effective genetic manipulation has been performed to explore functional proteins in *P. xylostella*, especially exogenous gene products of PDV that integrate into the host genome after embryonic development. In this study, RNAi was chosen as the optimal system for testing the effect of virulence factors. Although gene knockdown is difficult to achieve in Lepidoptera, the effectiveness of PDV gene interference is relatively high, with a 70–80%, and sometimes a 90%, decrease in target gene expression [60,61]. In addition, it is a technical challenge to overexpress genes in Lepidoptera, and previous studies have used baculovirus as a transient expression system to analyze CvBV-gene cellular immune responses [40,62,63]. Therefore, we took advantage of the Bac-to-Bac baculovirus expression system, which enables the rapid and efficient expression of target genes. NPV was injected into the *P. xylostella* larvae and infected the tissues, leading to the expression of the CvBV_12-6 protein. To exclude the effect of baculovirus infection on *P. xylostella*, we strictly examined the response of hemocytes within 24 h, in which CvBV_12-6 was highly expressed, and there were no obvious symptoms caused by baculovirus infection. Additionally, the negative control NPV-GFP was an important reference for the interpretation of the results. Taken together, the results of this study show evidence for the potential function of bracovirus PTPs.

There are some commonalities and differences among the function of *CvBV_12-6* in *D. melanogaster* and *P. xylostella*. The difference is that *CvBV_12-6* did not change the viability of hemocytes in *D. melanogaster*, but impaired that in the host *P. xylostella*, which may have been a result of the genetic difference between these two species. Concerning a decrease in host hemocyte viability, some BV PTPs have been shown to induce apoptosis in the *Sf21* cell line via caspase activation [39]. However, this does not seem to be the cause for *CvBV_12-6* because the total number of host hemocytes was not affected. Cell death and survival involve multiple processes; therefore, more experiments are needed to explore the role of CvBV_12-6 in inducing host cell death. *CvBV_12-6* inhibited melanization reactions in both insects, consistent with early findings after *C. vestalis* parasitization [55,64] and CvBV injection [55]. Although the mechanism via which *CvBV_12-6* regulates host melanization remains unclear, the regulation of phosphorylation levels during melanization in *D. melanogaster* has been extensively studied. In *D. melanogaster*, p38 MAPKs are preferentially activated in response to a wide variety of stress stimuli, and thereafter phosphorylate various substrates to regulate cellular immune and stress responses [65,66,67]. Sekine et al. found that p38 functions in the dopamine synthesis pathway to activate the melanization reaction and may be involved in immune and stress responses [68]. Therefore, we propose that *CvBV_12-6* may inhibit melanization by negatively regulating the MAPK pathway or directly suppressing the survival of hemocytes in the *P. xylostella* host. Furthermore, our previous studies have shown that CLP genes in CvBV [55] and a trypsin inhibitor-like protein [69] in teratocytes functioned as melanization inhibitors, indicating multistep regulation of the host PO pathway mediated by parasitic wasp-associated factors.

In summary, we identified a PTP gene in *C. vestalis* BV, *CvBV_12-6*, that showed tyrosine phosphatase activity. *CvBV_12-6* inhibited both the cellular and humoral immunity of *P. xylostella* by reducing the hemocyte viability or suppressing phenoloxidase/melanization responses individually, and it also showed functional immunosuppression in the ectopic host *D. melanogaster.* Our results not only provide evidence for an inhibitory role played by a newly characterized gene family (PTP) in the host melanization response, but also expand our knowledge about the mechanisms by which parasitoids regulate the humoral immunity of their hosts.

## Figures and Tables

**Table 1:** The primer sequences for functional studies of CvBV-PTP24.

Primer Name	Primer Sequence (5′ to 3′)	Purpose
PTP24-orf-SP	AAATGAGTTCTAACAAGGCG	gene cloning
PTP24-orf-AP	TGTTGTTCCCAGACCATTTTCC	gene cloning
PTP24-qrt-SP	TCGACAGCTTCAAACAACCC	qPCR
PTP24-qrt-AP	GTCGCCCGTTCTTCCAATT	qPCR
Px-β-tubulin-SP	GACGCATGTCCATGAAGGAG	qPCR
Px-β-tubulin-AP	CCAATGCAAGAAAGCCTTGC	qPCR
PTP24-BamHI-SP	CGCGGATCCATGAGTTCTAACAAG	eukaryotic expression
PTP24-XhoI-AP	CCGCTCGAGTTACGTATAAAGATTC	eukaryotic expression and transgenic fruit fly
PTP24-NotI-SP	ATTTGCGGCCGCATGAGTTCTAACAAG	transgenic fruit fly
PTP24-dsRNA-SP	GGTCTGGGAACAACAATCTG	RNAi
PTP24-dsRNA-AP	TCTCAGTTGGGACACGATAG	RNAi
PTP24-dsRNA-SP-T7	TAATACGACTCACTATAGGGGTCTGGGAACAACAATCTG	RNAi
PTP24-dsRNA-AP-T7	TAATACGACTCACTATAGGTCTCAGTTGGGACACGATAG	RNAi
GFP-dsRNA-SP	CAGTGCTTCAGCCGCTACCC	RNAi
GFP-dsRNA-AP	CTTCTCGTTGGGGTCTTTGCT	
GFP-dsRNA-SP-T7	TAATACGACTCACTATAGGCAGTGCTTCAGCCGCTACCC	RNAi
GFP-dsRNA-AP-T7	TAATACGACTCACTATAGGCTTCTCGTTGGGGTCTTTGCT	RNAi

## Data Availability

Not applicable.
